# An auxin-inducible degron system for conditional mutation in the fungal meningitis pathogen *Cryptococcus neoformans*

**DOI:** 10.1093/g3journal/jkaf071

**Published:** 2025-04-07

**Authors:** Manning Y Huang, Matthew J Nalley, Patrick C Hecht, Hiten D Madhani

**Affiliations:** Department of Biochemistry and Biophysics, University of California San Francisco, San Francisco, CA 94158, USA; Department of Biochemistry and Biophysics, University of California San Francisco, San Francisco, CA 94158, USA; Department of Biochemistry and Biophysics, University of California San Francisco, San Francisco, CA 94158, USA; Department of Biochemistry and Biophysics, University of California San Francisco, San Francisco, CA 94158, USA

**Keywords:** *Cryptococcus neoformans*, auxin-induced degron, essential genes, FKS1

## Abstract

*Cryptococcus neoformans* is the top-ranked W.H.O. fungal priority pathogen, but tools for generating conditional mutations are limited. Auxin-inducible degron systems permit rapid and effective cellular depletion of a tagged protein of interest upon adding a small molecule. These tools are invaluable, particularly for studying essential genes, which may play important roles in pathogen biology. AID2 is one such system that improves on previous strategies. This system achieves greater sensitivity and specificity through an auxin derivative, 5-Ph-IAA, alongside an OsTIR1*_F74G_* mutant. We adapted the AID2 system for *C. neoformans* by codon optimizing OsTIR1*_F74G_* and tested its use in multiple scenarios. We demonstrate that the *C. neoformans* optimized AID2 system enables effective degradation of proteins, including essential proteins, and can be used to help discriminate essential from nonessential genes. This tool enables the study of unexplored parts of the *C. neoformans* genome.

## Teaser

This paper described the adaptation of an auxin-induced degron system (AID2) to the human fungal pathogen *Cryptococcus neoformans* including characterization of three different degron tags. This enables the conditional depletion of any protein, including those encoded by essential genes. A suite of plasmids and strains have been generated and made available to the community.

## Introduction

*Cryptococcus neoformans* is an opportunistic fungal pathogen of significant clinical importance (Rajasingham *et al*. 2017; Fisher and Denning 2023). Our understanding of fungal pathogens has been accelerated by the development of tools for the genetic manipulation of fungal species (Ross and Santiago-Tirado 2024). In *C. neoformans*, a genome-wide deletion collection has permitted the high-throughput dissection of gene function in pathogenesis and basic biological pathways (Boucher *et al*. 2024). While this collection contains 4,328 high-confidence deletion mutants, deletions were not obtained for 2,523 genes, representing 36% of the *C. neoformans* proteome. One prominent explanation for an inability to disrupt a given gene of interest is that the gene of interest may be essential. While some proportion of essential genes represent well-conserved pathways, many of these putatively essential genes are uncharacterized with no predicted orthology. As these nondisruptable genes ostensibly drive essential functions in an important human pathogen, additional tools are required for their study.

Controlling gene product expression is an invaluable tool for understanding how essential genes function. Most approaches rely on inhibiting RNA expression to decrease protein expression. This is commonly achieved by replacing the native promoter of a gene of interest with an exogenous promoter to achieve transcriptional repression. Within fungal pathogens, commonly used promoters include the Tet promoter (Tet-On/Tet-Off) and copper repressible promoters (Ory *et al*. 2004; Park and Morschhäuser 2005; Fu *et al*. 2024). In *C. neoformans*, Beattie and colleagues used the copper repressible promoter *P_CTR4_* to inhibit the essential gene *FKS1*, encoding the catalytic subunit of beta-glucan synthase (Beattie *et al*. 2022). Inhibition of expression with high copper led to increased cell wall chitin content in response to inhibition. In diploid *C. albicans*, the GRACE strain collection was constructed by systematic deletion of second allele and replacement of the promoter of the second allele with the Tet-Off promoter (Roemer *et al*. 2003). This collection was used to identify a partial set of *C. albicans* essential genes and identify genes and pathways involved in morphology switching (O’Meara *et al*. 2015). A Tet-Off system has recently been optimized for use in *C.* neoformans by Fu and colleagues and will be a promising tool for future systematic studies (Fu *et al*. 2024). While effective, promoter replacement strategies present a few disadvantages. Inducible/repressible promoters are known to have “leaky” expression or may overexpress genes under inducing conditions, causing misregulation of essential genes and confusing the interpretation of results. Some essential genes may also have low native RNA expression levels, and repressive promoters might not significantly decrease expression below those levels (Arita *et al*. 2021). Early methods to influence protein levels without affecting the native promoter include the DaMP system, which generated hypomorphic alleles by disrupting the 3′UTR of a gene of interest, preventing polyadenylation of mRNA and leading to mRNA instability and turnover (Muhlrad and Parker 1999; Schuldiner *et al*. 2005). The DaMP method was used to construct an essential gene hypomorph library in *S. cerevisiae*, and was used to study biofilm formation in *C. albicans* (Yan *et al*. 2008; Finkel *et al*. 2011). Other RNA inhibition-based strategies include RNAi and CRISPRi, which have been adapted to study fungal pathogens (Mouyna *et al*. 2004; Rappleye *et al*. 2004; Wensing and Shapiro 2022). However, RNA inhibition indirectly influences protein expression. Furthermore, changes in RNA expression are not strictly correlated with changes in protein expression and ultimately, inhibition efficiency will depend on protein stability and mRNA turnover (Cheng *et al*. 2018).

Directly targeting a protein of interest for degradation via the ubiquitin-proteasome pathway has recently become increasingly feasible with the development of new methods. These methods include proteolysis targeting chimeras, which use unique fusion proteins to recruit the E3 ligase complex to a tagged protein of interest (PROTACs); dTAGs, which add versatility to PROTACs by using a standardized chimera to target a protein of interest fused with an FKBP12 subunit; and auxin-inducible degrons, which behave similarly but use simpler components (Nishimura *et al*. 2009; Nabet *et al*. 2018; Prozzillo *et al*. 2020). In fungal pathogens, auxin-inducible degron systems have been developed for use in *C. albicans* and *C. glabrata* but have yet to be adapted for use in *C. neoformans* (Milholland *et al*. 2023). Additionally, the development of electroporation-based CRISPR-Cas9 techniques has improved on older biolistics technology for transformation and has made tag-based tools easier to use (Fan and Lin 2018). We previously demonstrated that short homology arms could be used with a *C. neoformans* codon-optimized copy of Cas9, permitting tagging genes of interest with as little as 50 bp of homology (Huang *et al*. 2022). In this study, we converted and tested the auxin-degron AID2 system in *C. neoformans* and demonstrated that this system is robust and tunable to deplete proteins of interest.

## Methods

### Media and growth conditions

*Cryptococcus neoformans* strains were routinely grown in liquid YPD media [2% w/v Bacto Peptone (#211820, Gibco), 2% w/v dextrose, 1% w/v yeast extract (#212720, Gibco)] or on solid YPD media with 2% w/v agar. Where specified, *C. neoformans* strains were also grown in YNB media [0.15% w/v yeast nitrogen base powder without ammonium sulfate (#4027032, MP Biomedicals), 0.5% ammonium sulfate, 2% w/v dextrose]. Transformations using amdS selection were plated on YNB without ammonium sulfate plates supplemented with 5 mM acetamide. Liquid cultures were grown at 30°C in a roller drum incubator set to 60 RPM. Cultures grown on solid media were normally incubated at 30°C for 2–3 days unless otherwise specified. To save *C. neoformans* strains, glycerol was added to cells taken from fresh liquid cultures to a final concentration of 15–20% v/v, then stored at 80°C.

5-Ph-IAA (#HY-134653, Med Chem Express) was ordered as solid powder and resuspended to 10 mM in DMSO for stock solution. 5-Ph-IAA stocks and dilutions were kept at −20°C.

A list of strains, primers, and plasmids used in this study can be found in Supplementary Table 1.

### Plasmid cloning

Plasmids were generated by gap repair in *S. cerevisiae*. Briefly, linearized pRS316 vector and DNA products with overlapping homology were transformed into *S. cerevisiae* strain BY4741 using the lithium acetate and ssDNA carrier DNA protocol (Sikorski and Hieter 1989; Gietz and Schiestl 2007). *S. cerevisiae* transformants were plated on YNB plates lacking uracil and plasmid DNA was recovered from yeast transformant cells using the Zymoprep Yeast Plasmid Miniprep II Kit (D2004, Zymo Research), and then transformed into DH5α *Escherichia coli* cells by electroporation. Plasmid DNA was recovered from *E. coli* using Nucleospin Plasmid Mini kits (#740588.50, Macherey-Nagel). All plasmids were sequenced by whole-plasmid sequencing performed by Plasmidsaurus using Oxford Nanopore Technology with custom analysis and annotation. All PCRs described in this section were performed using Q5 High-Fidelity Polymerase (#M0491, New England Biolabs).

DNAs containing codon-optimized *OsTIR _F74G_* and *mAID* were ordered from Genscript in vector format. DNAs containing codon-optimized mIAA7 and unoptimized AID* were ordered from IDT as gBlock HiFi Gene Fragments. Two cloning steps were used to assemble each of the plasmids pBHM2461-2463. In the first step, *OsTIR _F74G_* was cloned into pRS316 alongside *TEF1* promoter and terminator, as well as a neomycin resistance marker, yielding pBHM2421. This vector also contained additional components that were not ultimately used. Seven PCR products with overlapping homology were used in total, and the primers and templates used for each product are listed in Supplementary Table 1 (Sikorski and Hieter 1989; Arras *et al*. 2015; Erpf *et al*. 2019; Huang *et al*. 2022). In the second round, *OsTIR1_F74G_* and the *NEOR* marker were amplified from pBHM2421 and cloned into pRS316 with t2a-mNeonGreen amplified from *BLP2:t2a:mNeonGreen C. neoformans* strain CM2034 (Matt Nalley, unpublished data). Four PCR products with overlapping homology were used for this cloning step.

pBHM2426 carries a NATR marker and an empty sgRNA with a BplI cut site between the *C. neoformans U6* promoter and scaffold (Manning Huang, unpublished data). Plasmids pBHM2644-6 were cloned by inserting 2 PCR products into pBHM2426 in a single step. Primers and products used are listed in Supplementary Table 1. Note that P23 was paired with one of either P24-26, depending on the degron amplified.

Although the BplI site in pBHM2644-6 was not used for this work, an sgRNA may be cloned into this site. Briefly, 60 bp ssDNA oligos containing a desired sgRNA sequence and flanking homology can be ordered. This oligo may then be directly cloned into BplI digested pBHM2644-6 using an NEB HiFi DNA Assembly Kit (#E2621, New England Biolabs) per manufacturer's instructions to bridge dsDNA with a ssDNA oligo.

**Fig. 1.**
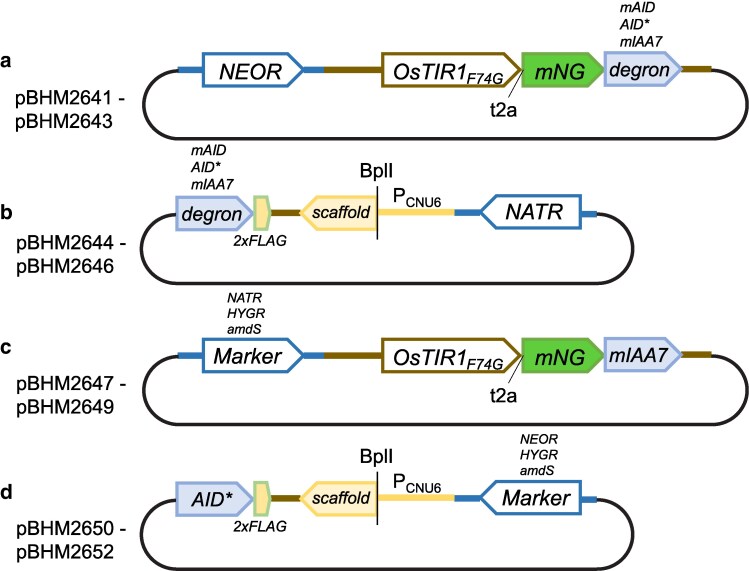
Plasmid maps a) Plasmid series pBHM2641 through BHM2643 carry *OsTIR1_F74G_-t2a-mNG*-*degron* marked with a neomycin resistance marker for selection and are used to introduce *OsTIR1 _F74G_* at the Safe Haven locus by insertion. Each plasmid carries a different degron, either mAID, AID*, or mIAA7. b) Plasmid series pBHM2644 through BHM2646 carry only a degron tag (either mAID, AID*, or mIAA7) followed by a 2×FLAG epitope tag, an empty sgRNA, and a nourseothricin resistance marker for transformant selection. These vectors are used as PCR templates to generate PCR products to degron tag a gene of interest. c) Plasmid series pBHM2647 through BHM2649 are provided for additional selectable marker options and carry *OsTIR1 _F74G_-t2a-mNG*-*mIAA7* but with either the *NATR, HYGR,* or *amdS* markers. d) Plasmid series pBHM2650 through BHM2652 carry the AID* degron, empty sgRNA, and either *NEOR*, *HYGR*, or *amdS* markers to act as PCR templates for AID* tagging a gene of interest.

In order to increase the flexibility of using the *C. neoformans* AID2 degron system for tagging, we have additionally generated versions of pBHM2642 with each of the markers *NATR*, *HYGR*, or *amdS* instead of *NEOR* (Fig. 1c). We have also generated versions of pBHM2644 carrying *HYGR*, *NEOR*, or *amdS* instead of *NATR* (Fig. 1d). To generate these vectors, the *NEOR* or *NATR* marker was cut out of pBHM2642 or pBHM2644, respectively, and alternative markers were amplified using primers P54 and P55. The PCR product was then cloned into pBHM2642 or pBHM2644, as appropriate, by gap repair in *S. cerevisiae*, then validated as described above.

### Strain manipulation

Genetic manipulations of *C. neoformans* strains were performed by electroporation as described elsewhere (Lin *et al*. 2020). Briefly, log phase cells were washed in cold water and treated with 1 mM DTT in electroporation buffer (10 mM Tris-HCl pH 7.5, 1 mM MgCl_2_, 270 mM sucrose) to render cells electrocompetent. DNA was then added to the cells, transferred to a 0.2 cm gap Gene Pulser Electroporation Cuvette (#1652089, Bio-Rad) and electroporated in a Gemini X2 Twin Wave Electroporator (#45-2040, BTX Molecular Delivery Systems) with the following settings: 500 V, 400 Ω, 250 μF. Although we used this specific model, electroporators have easily succeeded with different models but under different optimized settings.

Following electroporation, cells were recovered for 2 h in liquid YPD media at 30°C with shaking, then plated on solid YPD media supplemented with selective drugs. Final concentrations of either 125 ng/μL nourseothricin (#N51200, RPI) or 200 ng/μL G418 (#61-234-RG, Corning) were used as appropriate.

Primers used to generate sgRNA-expressing DNA and donor DNA are listed in Supplementary Table 1. sgRNA cassettes used in this study were amplified using fusion PCR as previously described using pBHM2329 as a template (Huang *et al*. 2022). All PCRs for this section were run using ExTaq DNA polymerase (#RR001, Takara Bio), cleaned as previously described using Nucleospin Gel and PCR Clean-up kits (#740609, Macherey-Nagel), and eluted in water for electroporation into *C. neoformans* cells.

CM2473-5 were generated by transforming CM2049 with linearized pBHM2641-3, respectively without homology arms, as well as SH1 sgRNA expressing PCR product to insert *OsTIR1 _F74G_* into the *SH1* locus. CM2476 was generated by transforming CM2474 with *amdS* marker amplified from pPEE7 (Erpf *et al*. 2019) without homology arms and YKU80 sgRNA expressing PCR product to disrupt *YKU80*. CM2473 served as the parental strain to each essential gene degron tag strain. To generate each degron-tagged essential gene strain CM2477-93 used in this study, 2 PCR products were used, one specifying an sgRNA targeting the downstream region of the gene to be degron-tagged and one carrying the degron tag and *NATR* marker. PCRs to amplify degron and NATR marker were run using long oligos with overhangs to provide homology arms. All transformants were verified by colony PCR and PCR genotyping using genomic DNA. Genomic DNA was isolated as previously described using the *C. neoformans* version of the “smash and grab” protocol (Lin *et al*. 2020). Primers used for PCR genotyping are listed in Supplementary Table 1 (P29, P42-53). Two sets of primers were used for genotyping—one to check for the presence of the tag allele and a second to check for the absence of the wild-type allele. To validate *RSA4* junctions by Sanger sequencing, the tag junction was amplified using P29 and P45 then sequenced by Genewiz using either P29 or P45 as a sequencing primer.

### Flow cytometry

To prepare samples for flow cytometry, *C. neoformans* cells were inoculated in liquid YNB media and grown to OD 0.5. Cells were then split into 3 culture tubes, and either DMSO was added or 5-Ph-IAA was added to a final concentration of 1 μM or 100 nM. Samples were taken from each culture at each time point and were diluted to OD 0.05 with fresh YNB. One milliliter of the diluted sample was transferred to a round-bottom FACS culture tube and loaded into a BD Accuri C6 Plus. The BD Accuri C6 Plus uses a 488 nm laser with a 533/30 bandpass filter. 10,000 events were acquired per sample on medium speed setting. Events data were analyzed using the Python FlowCal library (version 1.3.0) (Castillo-Hair *et al*. 2016).

### Western blot analysis

The same samples used for flow cytometry were also used for western blot analysis. Cells were processed as described previously (Boucher *et al*. 2024). Volumes equivalent to 2 ODs were taken at each time point from mNG-AID* and mNG-mIAA7 samples. Cells were washed in water and then resuspended in 10% TCA and incubated on ice. Samples were pelleted at 21,000 g at 4°C then washed with cold acetone and pelleted again. Pellets were then air-dried and resuspended in a final concentration of 2× NuPAGE LDS sample buffer (#NP0007, Invitrogen), 50 mM Tris-HCl pH 8.0, and 100 mM DTT along with 0.5 mm zirconia/silica beads (#11079105Z, BioSpec). Samples were bead-beaten using an Omni Bead Rupter Elite with the following settings: (2 × 90 s cycles, 6.0 m/s, 90 s rest). Samples were then separated from zirconia beads by centrifugation and saved at −70°C for later western blot analysis.

Before loading, samples were boiled and then run on Surepage 4–12% Bis-Tris gels (#M00653, GenScript). Samples were then transferred onto 0.45 um nitrocellulose membranes in Tris-Glycine transfer buffer (25 mM Tris, 250 mM glycine) with 20% methanol. Membranes were blocked for 1 h in 5% milk in TBST (10 mM Tris-HCl pH 7.4, 150 mM NaCl, 0.1% Tween-20). For primary antibody, membranes were first blotted using 1:1500 ChromoTek mouse-anti-mNeonGreen monoclonal antibody (#32F6, ProteinTech) in blocking buffer overnight at 4°C. Membranes were then washed 3 times using 15 min incubations in TBST. Membranes were then incubated for 1 h at 25°C in 1:8000 goat-anti-mouse IgG (#31430, Invitrogen) HRP-conjugated secondary antibody. Membranes were washed another 3 times in TBST then visualized using SuperSignal West Pico Plus chemiluminescent substrate (#34580, Thermo Fisher Scientific). Afterwards, membranes were stripped using Restore Western Blot Stripping Buffer (#21059, Thermo Scientific) per manufacturer's instructions, washed, then steps above were repeated using 1:1000 rabbit-anti-Histone H3 polyclonal antibody (#65-6120, Invitrogen) as primary antibody and 1:8000 goat-anti-rabbit IgG (#65-6120, Invitrogen) as secondary antibody to visualize H3 as a loading control.

For Trr1-degron-flag or Erg-degron-flag westerns shown in Fig. 3c, western blots were performed as described above except using 1:3000 mouse-anti-FLAG (#F3165, Sigma-Aldrich) as primary antibody. Cas9 in these strains carries an HA tag, and was used as a loading control. Cas9-HA was probed using anti-HA-peroxidase (#12013819001, Sigma-Aldrich) as primary antibody without an additional secondary antibody.

### Spotting and growth assays

To prepare cells for spotting assays, strains were grown overnight in YPD. For spotting assays, cells were diluted to OD 0.3 in fresh YPD. Five-fold serial dilutions were taken from this culture for a total of 6 dilutions ranging from OD 0.3 to OD 9.6E-5. Three microliter of each dilution was spotted onto YPD plates with or without 1 μM 5-Ph-IAA. This roughly corresponds to an expected 9,000 down to 3 CFUs per spot from highest to lowest dilution. Cells were grown for 2 days at 30°C then imaged and allowed to continue to grow at room temperature before being imaged again.

For liquid growth assays, degron-tagged strains were first grown overnight in YPD, then serially diluted to OD 0.02 in fresh YPD. 50 μL of YPD with 2× final 5-Ph-IAA concentration to be tested was added to wells of a 96-well untreated round-bottom plate (#229590, CELLTREAT). 50 μL of OD 0.02 cells were added to each well except blank control wells. The plate was then covered with a Breathe-Easy polyurethane film (#9123-6100, USA Scientific) following which cells were grown and ODs were measured every 15 min across 48 h in a Tecan Infinite 200 Pro with the following settings: 30°C incubation, orbital shaking with 4 mm amplitude. At least 3 replicates for each degron tagged strain were grown in each 5-Ph-IAA concentration. More than 3 replicates were regularly grown as the polyurethane film over some wells could be breached during experimental handling, and data from these wells was omitted from analysis. Growth curve data were analyzed and relative fitness was predicted using the Python package Curveball (v0.2.16) (Ram *et al*. 2019). Statistical analysis was performed using GraphPad Prism software (v10.4.1).

## Results

### Design and expression optimization

**Fig. 2.**
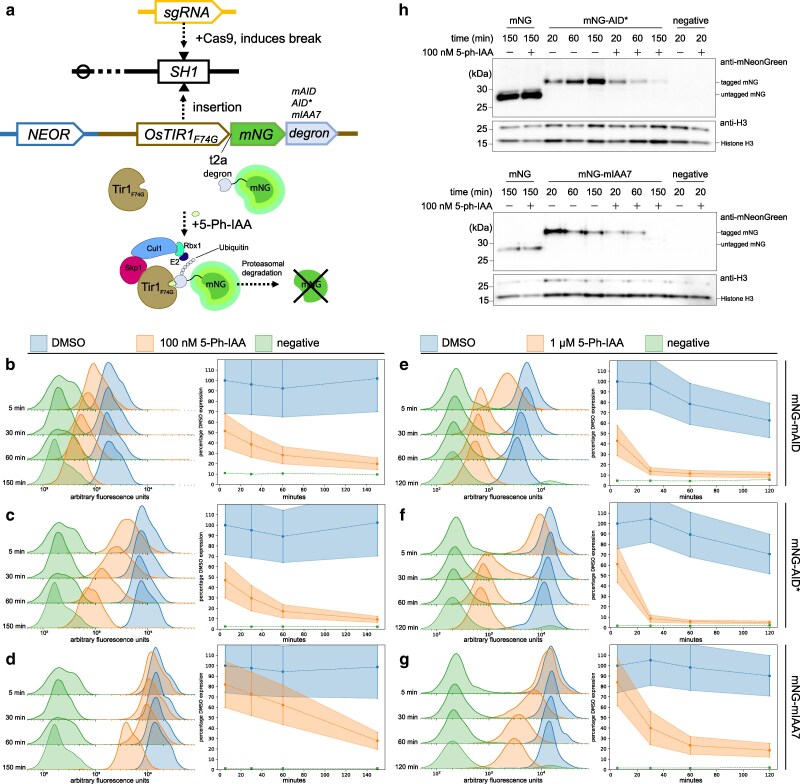
Testing mNeonGreen degradation a) Experimental scheme showing insertion of *OsTIR1_F74G_-t2a-mNG-degron* into the SH1 locus and expected degradation of mNG in the presence of 5-Ph-IAA. b) Flow cytometry data showing decrease in mNeonGreen signal across multiple timepoints after treatment of an mNG-mAID expressing strain with 100 nM 5-Ph-IAA. Overlapping histograms in left panel show fluorescence signal in arbitrary fluorescence units for different populations of cells. Blue histograms show DMSO treated cells, orange histograms are cells treated with 100 nM 5-Ph-IAA, and green histograms are WT cells not expressing mNG. Each set of 3 histograms correspond to an axis, each axis corresponds to a timepoint—either 5, 30, 60, or 150 min for b–d), or after 120 min for e–g). Right panel summarizes histogram data into a line graph with bars showing IQR with time in minutes on the *x*-axis. Data are presented as a percentage of median DMSO mNG expression at the 5 min timepoint. c) Same as b) except for mNG-AID*. d) Same as b) except for mNG-mIAA7. e) Same as b), except cells were treated with 1 μM 5-Ph-IAA. f) Same as b), except for mNG-AID* treated with 1 μM 5-Ph-IAA. g) Same as b), except for mNG-mIAA7 treated with 1 μM 5-Ph-IAA. h) Western blot analysis of mNG signal across multiple timepoints using anti-mNG. Loading controls using anti-H3 are depicted in smaller panels with band corresponding to Histone H3 labeled.

We set out to create a toolbox for facile inducible protein degradation in *C. neoformans* (Fig. 1). We selected the improved AID2 system for this purpose because AID2 is anticipated to cause less leaky degradation and to degrade degron-tagged proteins more completely (Yesbolatova *et al*. 2020). This system uses an F74G mutant of the Oryza sativa F-box auxin receptor gene *TIR1* (Yesbolatova *et al*. 2020). When OsTir1 binds auxin, it forms an E3 ligase complex, which ubiquitinates any proteins carrying a specific degron tag, leading to degradation of the protein of interest through the proteasomal degradation pathway (Fig. 2a). To implement this system, we synthesized a *C. neoformans* codon-optimized version of *OsTIR1_F74G_* using the codon optimization scheme we previously employed for Cas9 expression (Huang *et al*. 2022). As introns are required for efficient gene expression in *C. neoformans*, we also included the same efficiently spliced intron we previously used for *CAS9* expression in the optimized *OsTIR1_F74G_* gene (Huang *et al*. 2022). Previous work in several species has shown that different degron tags can be degraded with varying efficiency (Li *et al*. 2019; Sepers *et al*. 2022). Therefore, to maximize degradation efficiency, we chose to test 3 commonly used degron tags: mAID, mIAA7, and AID*. The mAID and mIAA7 tags, but not the AID* tag, were also optimized for expression using our *C. neoformans* codon optimization scheme.

**Fig. 3.**
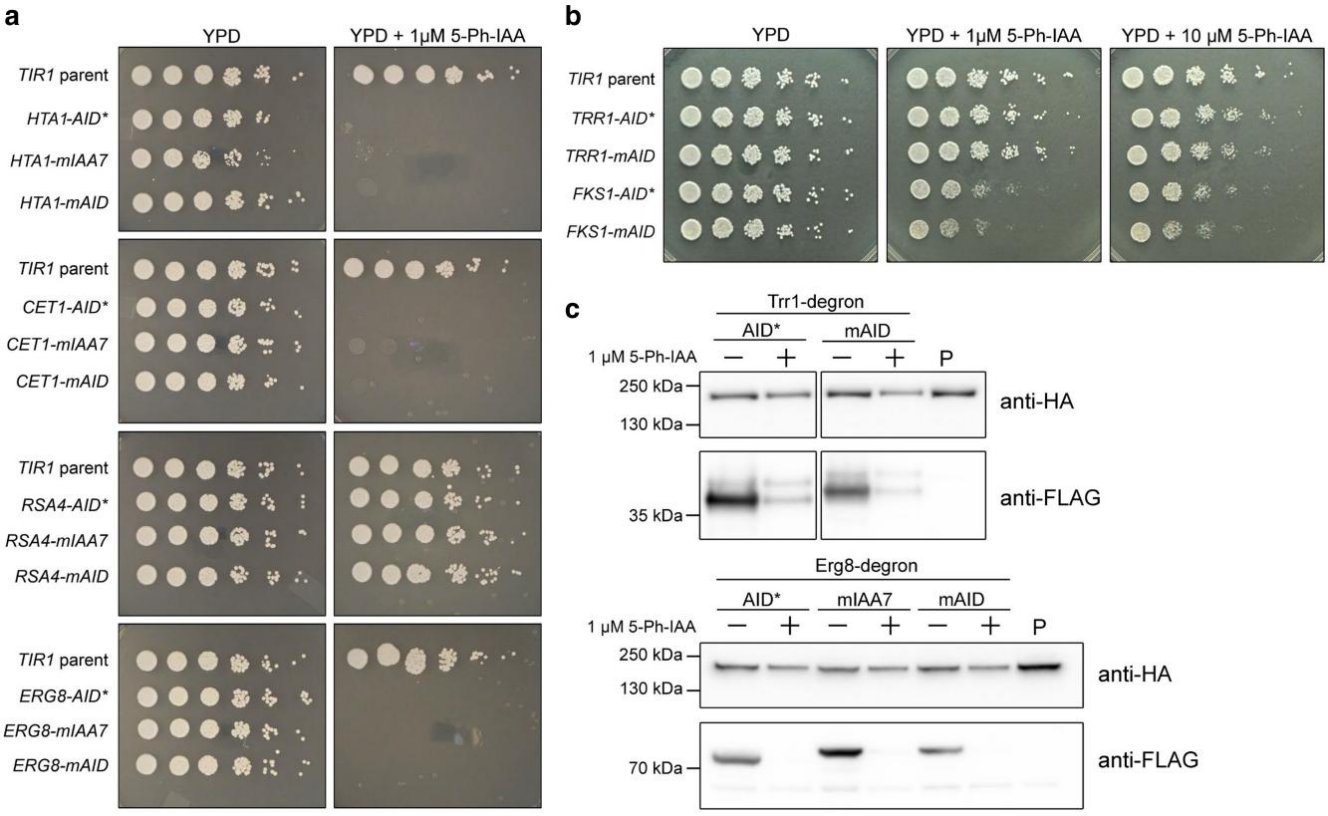
Spotting assays for degron tagged essential genes a) *HTA1*, *CET1*, *RSA4*, or *ERG8* tagged with either mAID, AID*, or *mIAA7* degrons were spotted across a 5-fold serial dilution onto YPAD solid media with or without 1 μM 5-Ph-IAA. Cells were grown for 2 days at 30°C then imaged. b) *TRR1* or *FKS1* tagged with either AID* or *mAID* degrons were spotted as in (a) onto either YPD solid media without 5-Ph-IAA, YPD + 1 μM 5-Ph-IAA, or YPD + 10 μM 5-Ph-IAA. Cells were grown for 2 days at 30°C then imaged. c) Western blots of *TRR1* or *ERG8*-degron tagged strains grown in liquid YPD with or without 1 μM 5-Ph-IAA at 30°C for 6 h. *mAID*, AID*, and *mIAA7* degron tags carry a 2×FLAG epitope tag and Trr1-degron or Erg8-degron were probed using anti-FLAG. The parental strain (sample labeled P) expressing *TIR1-t2a-mNG-mIAA7* also expresses Cas9-HA under the control of a strong constitutive *TEF1* promoter. Anti-HA was used as a loading control for all samples.

To test if an optimized AID2 system could degrade degron-tagged proteins of interest, we cloned 3 constructs using the constitutive *TEF1* promoter to drive expression of *OsTIR1_F74G_* followed by a t2a ribosomal skip sequence connected to *mNeonGreen* (*mNG*) tagged with one of the above codon-optimized degrons (Fig. 1a). Each construct also carries a G418/neomycin resistance marker for drug selection. We used Cas9 to insert these constructs into the *SH1* locus, and employed flow cytometry to assess fluorescence after treatment of cells with 5-Ph-IAA or DMSO (Arras *et al*. 2015). In the absence of 5-Ph-IAA, strong fluorescent signal was detected from all 3 degron-tagged versions of mNG (Fig. 2b–g). We observed a relatively reduced signal from DMSO-treated cells carrying mNG tagged with mAID, suggesting that the mAID tag itself might affect mNG expression or destabilize the protein (compare blue DMSO peaks in Fig. 2b–g). However, consistent with expectations for correctly functioning degradation, the addition of 5-Ph-IAA to a final concentration of either 100 nM or 1 μM led to rapid loss of mNG signal for cells carrying any of 3 degron tags (Fig. 2b–g). While mAID and AID* tags appeared equally effective at inducing mNG degradation, reducing fluorescent signal to half maximum within 5 min, mIAA7 tagged mNG expressing cells showed comparatively slower degradation. As loss of green fluorescent signal could also be explained by quenching of mNG by 5-Ph-IAA, whole cell extracts from AID* and mIAA7 tagged mNG were prepared from cells treated with 100 nM 5-Ph-IAA for western blot analysis using anti-mNG antibodies. We observed reduced band intensity, indicating decreased levels of mNG protein in samples treated with 5-Ph-IAA compared with untreated controls from cells tagged with either mIAA7 or AID* (Fig. 2h). These results indicate the *C. neoformans* optimized AID2 system functions robustly and can degrade a tagged protein of interest.

### Auxin-induced depletion of essential genes

Repeated failure to disrupt a gene of interest is a common approach to identifying essential genes (Akerley *et al*. 1998; van Opijnen *et al*. 2009; Segal *et al*. 2018). However, such an approach is fraught because it draws conclusions from negative data. Auxin-degron systems offer a useful orthogonal approach—the combination of a negative deletion result and inducible lethality or sickness creates a strong argument that a given gene is essential. We selected 6 genes for further analysis to test the optimized AID2 system in identifying essential genes. These correspond to *HTA1* encoding histone 2A*, CET1*, *TRR1*, *RSA4*, *ERG8,* and *FKS1* (standard genome names: *CNAG_06747*, *CNAG_06549*, *CNAG_05847*, *CNAG_04117*, *CNAG_06001,* and *CNAG_06508* respectively). Each of these genes except for *HTA1* has been reported as essential in the literature (Thompson *et al*. 1999; Missall and Lodge 2005; Ianiri and Idnurm 2015). *CET1*, *TRR1*, *RSA4*, *ERG8*, and *FKS1* are essential in *S. cerevisiae* (Giaever *et al*. 2002). *HTA1* may be nonessential in *S. cerevisiae* because 2 nearly identical copies exist and are synthetically lethal when both are deleted, while only one copy of *HTA1* exists in the *C. neoformans* genome (Kolodrubetz *et al*. 1982). *RSA4* essentiality might depend on strain background, but is likely nonessential in the laboratory KN99 background as we have been able to disrupt this gene in our efforts to generate a genome-wide deletion collection (Boucher *et al*. 2024). One potential limitation of auxin-degron systems is that membrane-bound or secreted proteins might be inaccessible to *OsTIR1* and may resultantly fail to be degraded. Previous reports have shown success in the depletion of membrane-bound proteins using the AID system in yeast and human cells (Nishimura *et al*. 2009; Li *et al*. 2019). These strategies successfully degraded membrane proteins by tagging proteins of interest on accessible cytoplasmic-facing termini (Nishimura *et al*. 2009). Inclusion of *FKS1* allows us to test this strategy with our system, as Fks1 is a large transmembrane protein with a cytoplasmic-facing C-terminus (Hu *et al*. 2023).

We generated plasmids carrying codon-optimized mAID, AID*, and mIAA7 (Fig. 1b). Each degron tag is preceded by a GSGSGGSG linker for C-terminal tagging and followed by a *2xFLAG* epitope tag for western blot analysis. This plasmid series also carries an empty sgRNA cassette, with the spacer sequence replaced by a BplI restriction enzyme site. Each plasmid also carries the *NATR* marker for drug selection. The intended use of this vector series is to allow cloning of sgRNAs into the BplI site using ssDNA oligonucleotides and Gibson assembly. However, for expediency, we used fusion PCR instead to separately amplify sgRNAs targeting downstream regions of our 5 genes of interest. Plasmids pBHM2644-6 were used as templates for PCR using primers with long overhangs to generate flanking short homology arms on donor DNA carrying the degron tag and *NATR* marker (See Methods). We used CRISPR-Cas9 to generate a *yku80* (encoding the nonhomologous end-joining factor Ku80) insertion mutant derivative from a *OsTIR1_F74G_-t2a-mNG-mIAA7* expressing strain to tag all 5 genes of interest in this strain. Goins and colleagues previously demonstrated that deletion of either *YKU70* or *YKU80* increases rates of homologous recombination in *C. neoformans* (Goins *et al*. 2006). We therefore decided to use a *yku80* mutant to increase the ease of tagging; we recently demonstrated that transforming a *yku80* disruption with donor and sgRNA cassettes on the same DNA fragment routinely achieves 99%+ efficiency in tagging (Nalley *et al*. 2025). *TRR1-mIAA7* and *FKS1-mIAA7* tagged transformants were not obtained in a single attempt and were not constructed. All constructed strains were validated by colony PCR and PCR genotyping from genomic DNA.

We hypothesized that if a tagged gene was essential, then the tagged strain should fail to grow when degradation was induced by addition of 5-Ph-IAA. In the absence of 5-Ph-IAA, all strains grew similarly to wild-type on YPD media (Fig. 3). Independent of the degron version used, *CET1, ERG8, and HTA1* degron-tagged strains all failed to grow on YPD media supplemented with 1 μM 5-Ph-IAA after 48 h. Independent of the degron version used, *RSA4*-degron-tagged strains showed no growth defect on YPD+1 μM 5-Ph-IAA after 48 h. We confirmed the construction of our *RSA4*-degron-tagged strains by Sanger sequencing of the tag junction, and found the expected sequences for all 3 strains (Supplementary Fig. 1).

**Fig. 4.**
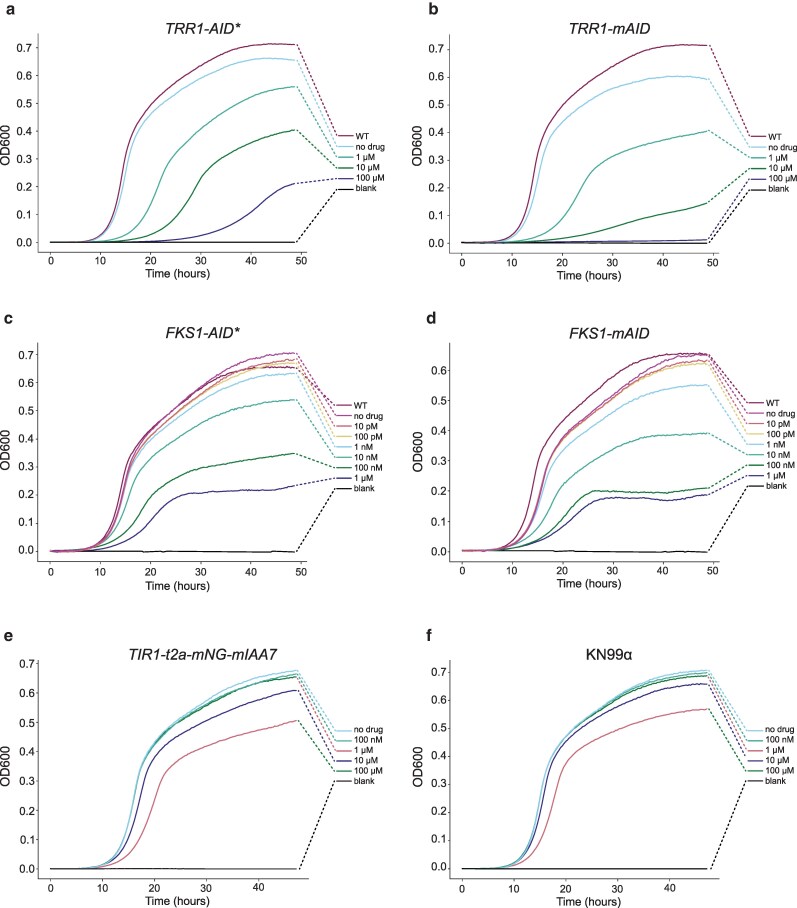
Response to depletion in liquid culture cells were grown in 96 well plates for 48 h at 30°C with shaking in YPAD supplemented with specified concentrations of 5-Ph-IAA. Growth curves show average OD600 measurements from at least 3 replicates with line coloring indicating the corresponding final concentration of 5-Ph-IAA. a) Growth curves for a TRR1-AID* expressing strain in either 0, 1, 10, or 100 μM 5-Ph-IAA, or parental strain grown in 1 μM 5-Ph-IAA (labeled Parent). b) Same as (a) except for *TRR1-mAID*. c) Growth curves for a FKS1-AID* expressing strain in either 0, 10, 100 pM, 1, 10, 100 nM or 1 μM 5-Ph-IAA, or parental strain grown in 1 μM 5-Ph-IAA (labeled Parent). d) same as (c) except for *FKS1-mAID*. e) *TIR1-t2a-mNG-mIAA7* expressing strain grown in either 0, 100 nM, 1, 10, or 100 μM 5-Ph-IAA. f) Same as in (e) except for KN99α.

For *TRR1* and *FKS1*, only partial growth defects were observed under depletion conditions (Fig. 3b). *TRR1* degron-tagged strains showed only a subtle difference in colony size when grown on YPD+10 μM 5-Ph-IAA but not on 1 μM 5-Ph-IAA or without drug. We therefore asked whether Trr1 protein was being degraded in the presence of 5-Ph-IAA. Western blot analysis of TRR1-AID* and *TRR1-mAID* strains showed that Trr1 protein was successfully depleted in the presence of 1 μM 5-Ph-IAA, but depletion appeared incomplete as compared to *ERG8-degron* strains where tagged protein was not visible in the presence of 1 μM 5-Ph-IAA (Fig. 3c). These data suggest that *C. neoformans* may be viable with even relatively low amounts of Trr1 protein. Higher concentrations of 5-Ph-IAA may drive more complete depletion. We therefore grew *TRR1 degron* strains in liquid YPD media supplemented with 1, 10, or 100 μM 5-Ph-IAA. Growth defects for TRR1-mAID and TRR1-AID* expressing strains were more evident under liquid culture conditions (Fig. 4a and b). TRR1-AID* showed a significant difference in relative fitness at 10 μM 5-Ph-IAA, while *TRR1-mAID* showed a significant difference in relative fitness at 1 μM 5-Ph-IAA and complete inhibition of growth when treated with 100 μM 5-Ph-IAA (Fig. 4b, Supplementary Fig. 2a). While only one independent transformant was tested for TRR1-AID* and *TRR1-mAID*, these data further highlight that depletion efficiency may depend on the degron version used and the tagged gene of interest.

*FKS1* tagged with either *mAID* or AID* showed a clear but incomplete growth defect when grown on YPD supplemented with 1 μM 5-Ph-IAA or 10 μM 5-Ph-IAA but not without drug (Fig. 3b). These results demonstrate that even membrane-bound proteins in *C. neoformans* may be successfully degraded by AID2. The incomplete growth defect suggests that *C. neoformans* may tolerate low levels of Fks1, in line with previous work by Beattie and colleagues that shows *C. neoformans* may tolerate a 100-fold reduction in RNA expression with only a modest growth defect (Beattie *et al*. 2022). Taken together, these results highlight the importance of using a depletion-based approach as one of multiple orthogonal approaches to establish gene essentiality.

While protein depletion systems used in this manner permit the identification of essential genes, their primary application is to allow the study of conditional mutant phenotypes. To test this feature, we asked if we could ameliorate the *FKS1* growth defect by reducing the concentration of 5-Ph-IAA used. We grew AID* and *mAID* tagged versions of *FKS1* in liquid YPD media at 30°C across decreasing concentrations of 5-Ph-IAA. Both FKS1-AID* and *FKS1-mAID* tagged versions of *FKS1* showed significantly reduced growth when compared with untagged or DMSO treated cells (Fig. 4c and d, Supplementary Fig. 2b and c). Additionally, for both degron tags, growth rate appeared to follow a dose-responsive relationship between 1 nM and 1 μM 5-Ph-IAA. As little as 100 nM 5-Ph-IAA for FKS1-AID* and 10 nM 5-Ph-IAA for *FKS1-mAID* were able to induce significant decreases in relative fitness. These results highlight the robustness and tunability of auxin-degron systems in generating conditional mutants for study.

### 5-Ph-IAA toxicity

One driving factor for development of the AID2 system was ligand toxicity of the auxin indole-3-acetic acid (IAA) used by the first AID system (Yesbolatova *et al*. 2020). IAA is toxic to both *Saccharomyces cerevisiae* and mice at concentrations required to degrade degron-tagged proteins (Prusty *et al*. 2004; Suski *et al*. 2022). To test whether 5-Ph-IAA is toxic to *C. neoformans*, we grew wild type and an *OsTIR1_F74G_-t2a-mNG-mIAA7* expressing strain in liquid YPD media at 30°C across increasing concentrations of 5-Ph-IAA. In our hands, 100 μM 5-Ph-IAA is near the practical upper limit of auxin that can be used in liquid culture. Both strains showed a subtle but significant decrease in relative fitness at 100 μM 5-Ph-IAA but no significant difference in growth between treated and untreated cells was observed below this concentration (Fig. 4e and f, Supplementary Fig. 2d). As 100 μM 5-Ph-IAA is 100-fold higher than the normal dose, cells show only a subtle defect at this concentration of drug, and even nanomolar doses of 5-Ph-IAA can be sufficient to induce degradation of some genes, we believe 5-Ph-IAA toxicity should not be a concern in most cases.

## Discussion

This study presents a valuable addition to the growing *C. neoformans* genetic toolbox. We demonstrate that a *C. neoformans* codon-optimized AID2 degron system is effective for the rapid degradation of proteins of interest. Furthermore, we used the AID2 degron system to tag and deplete essential genes, demonstrating an approach to study essential gene function.

Establishing whether a gene is essential or not requires multiple orthogonal approaches and should avoid relying primarily on negative results. The genes examined in this study were motivated by at least 1 previous failure to delete that gene. Strains with the degron-tagged genes *HTA1*, *CET1*, *TRR1*, *ERG8*, and *FKS1* showed severe growth defects when treated with 5-Ph-IAA. The agreement between depletion experiments and failed deletion together presents a strong case for gene essentiality. We did not observe any phenotypic impact from the depletion of *RSA4*. *RSA4* was identified as an essential gene using segregant analysis and *Agrobacterium*-mediated mutagenesis (Ianiri and Idnurm 2015). However, Billmyre and colleagues identified *RSA4* as nonessential from genome-wide transposon mutagenesis experiments (Billmyre *et al*. 2024). Additionally, we were able to obtain an *rsa4* deletion mutant in our attempts to generate a *C. neoformans* genome-wide deletion library (Boucher *et al*. 2024). The disagreement between these results may be explained by differences in the strain background used in each of the above studies, but highlights the difficulty of relying on the failure to delete a gene alone to establish gene essentiality. For the same reason, while the combination of a successful deletion and lack of a depletion phenotype provides a strong case for *RSA4* nonessentiality, the depletion experiment would not be easily interpretable per se. The simplest explanations would be that we failed to degrade an essential gene sufficiently or that very little expression might be required for a wild-type growth phenotype. For difficult-to-degrade proteins, a combination of transcriptional and post-translational inhibition may provide more reliable control of gene function (Tanaka *et al*. 2015). Recent work by Fu and colleagues has established a Tet-Off system for *C. neoformans*, and the combination of this system with the AID2 system may result in more effective depletion of a gene of interest (Fu *et al*. 2024).

In addition to *Agrobacterium*-mediated mutagenesis, transposon mutagenesis, and systematic deletion of genes, analysis of meiotic progeny can be used to identify putative essential genes. An important recent development is the establishment of a stable homozygous diploid *C. neoformans* KN99 strain, CnLC6683 (Peterson *et al*. 2024). A heterozygous mutant can be constructed in this strain then sporulated on mating media. Failure to obtain a haploid spore carrying a deletion allele indicates the targeted gene may be essential. Alongside providing an orthogonal approach to establish whether a gene is essential or not, depletion strategies are particularly valuable in permitting the direct study of essential genes. In this context, studying haploinsufficiency phenotypes in a diploid strain can provide insight into gene function, but conditional mutants using a Tet-Off, *CTR4* promoter, or auxin-inducible degron approach may allow the study of any essential gene of interest. The responsiveness of AID2 to a wide range of 5-Ph-IAA dosage, as we have demonstrated with *FKS1 and* TRR1 degron-tagged strains is particularly useful in this context, as it allows an investigator to tune the strength of a phenotype to within a desired range.

Two critical advantages were described by Yesbolatova and colleagues in the development of the AID2 system—unlike AID, AID2 does not cause leaky degradation, and requires significantly lower concentrations of auxin to induce degradation (Yesbolatova *et al*. 2020). These advantages were clearly observed in our *C. neoformans* implementation, as degradation was not observed in the absence of 5-Ph-IAA, but could be induced with as little as 10 nM 5-Ph-IAA. We observed that the mAID tag caused destablization of mNG in our initial experiments. Furthermore, mAID and AID* degron tags behaved similarly but with noticeable differences for *FKS1* and *TRR1*. General wisdom accepts that polypeptide tags may behave idiosyncratically depending on gene and tag utilized. For this reason, we have presented 3 different tag versions: AID*, mAID, and mIAA7. Different marker combinations were also generated for integration of *OsTIR1_F74G_* (Fig. 1c) and tagging using the AID* degron (Fig. 1d). We broadly recommend that any *C. neoformans* degron experiments should try multiple degron tag versions for a gene of interest.

One desirable application for virulence studies may be to deplete a fungal protein of interest in an in vivo infection model. Unlike IAA, 5-Ph-IAA is not toxic to mice at concentrations required to induce protein degradation, and the literature reports no adverse effects were observed from intraperitoneal administration of as much as 10 mg/kg 5-Ph-IAA (Yesbolatova *et al*. 2020; Sladky *et al*. 2024). Interestingly, 5-Ph-IAA may be partially blocked by the blood-brain barrier, suggesting a possible application in niche-specific degradation of proteins of interest (Yesbolatova *et al*. 2020). While it is unclear how much auxin will be accessible to *C. neoformans* in a mouse host, our data suggest that even nanomolar concentrations of 5-Ph-IAA may induce sufficient degradation to observe depletion phenotypes. Additional experiments will be required to establish the feasibility of any in vivo studies using the AID2 degron system.

While this work utilized a *yku80* mutant to obtain tagged strains, tag strains can still be constructed using CRISPR-Cas9 and short homology arms as we have previously demonstrated. However, using a *yku80* strain ensures that nearly all transformants obtained are the correctly tagged strain of interest (Goins *et al*. 2006; Nalley *et al*. 2025). This approach opens the possibility of generating a genome-wide degron tag collection in either pooled or arrayed formats. We recently used pooled chemical-genetic interaction mapping to functionalize many previously uncharacterized genes, but this experiment was limited by the genes within the *C. neoformans* deletion collection (Boucher *et al*. 2024). A degron tag collection could include essential genes and genes with severely sick deletion phenotypes, increasing the mutant phenotypes we might observe and thereby improving the resolution of chemical-genetic interaction mapping. We anticipate that methods based on degron techniques will open interesting avenues of study in this important pathogen.

## Supplementary Material

jkaf071_Supplementary_Data

## Data Availability

Plasmids pBHM2641-pBHM2652 used in this study were deposited with Addgene. Strains are available upon request. Supplemental material available at G3 online.

## References

[jkaf071-B1] Akerley BJ, Rubin EJ, Camilli A, Lampe DJ, Robertson HM, Mekalanos JJ. 1998. Systematic identification of essential genes by in vitro mariner mutagenesis. Proc Natl Acad Sci U S A. 95(15):8927–8932. doi:10.1073/pnas.95.15.8927. 9671781 PMC21179

[jkaf071-B2] Arita Y, Kim G, Li Z, Friesen H, Turco G, Wang RY, Climie D, Usaj M, Hotz M, Stoops EH, et al 2021. A genome-scale yeast library with inducible expression of individual genes. Mol Syst Biol. 17(6):e10207. doi:10.15252/msb.202110207. 34096681 PMC8182650

[jkaf071-B3] Arras SDM, Chitty JL, Blake KL, Schulz BL, Fraser JA. 2015. A genomic safe haven for mutant complementation in *Cryptococcus neoformans*. PLoS One. 10(4):e0122916. doi:10.1371/journal.pone.0122916. 25856300 PMC4391909

[jkaf071-B4] Beattie SR, Jezewski AJ, Ristow LC, Wellington M, Krysan DJ. 2022. FKS1 is required for *Cryptococcus neoformans* fitness in vivo: application of copper-regulated gene expression to mouse models of *Cryptococcosis*. mSphere. 7(3):e0016322. doi:10.1128/msphere.00163-22. 35506343 PMC9241531

[jkaf071-B5] Billmyre RB, Craig CJ, Lyon J, Reichardt C, Eickbush MT, Zanders SE. 2024. Saturation transposon mutagenesis enables genome-wide identification of genes required for growth and fluconazole resistance in the human fungal pathogen *Cryptococcus neoformans*. bioRxiv 605507. 10.1101/2024.07.28.605507, preprint: not peer reviewed.

[jkaf071-B6] Boucher MJ, Banerjee S, Joshi MB, Wei AL, Huang MY, Lei S, Ciranni M, Condon A, Langen A, Goddard TD, et al 2024. Phenotypic landscape of a fungal meningitis pathogen reveals its unique biology. bioRxiv 619677. 10.1101/2024.10.22.619677, preprint: not peer reviewed.

[jkaf071-B7] Castillo-Hair SM, Sexton JT, Landry BP, Olson EJ, Igoshin OA, Tabor JJ. 2016. FlowCal: a user-friendly, open source software tool for automatically converting flow cytometry data from arbitrary to calibrated units. ACS Synth Biol. 5(7):774–780. doi:10.1021/acssynbio.5b00284. 27110723 PMC5556937

[jkaf071-B8] Cheng Z, Otto GM, Powers EN, Keskin A, Mertins P, Carr SA, Jovanovic M, Brar GA. 2018. Pervasive, coordinated protein-level changes driven by transcript isoform switching during meiosis. Cell. 172(5):910–923.e16. doi:10.1016/j.cell.2018.01.035. 29474919 PMC5826577

[jkaf071-B9] Erpf PE, Stephenson CJ, Fraser JA. 2019. *Amds* as a dominant recyclable marker in *Cryptococcus neoformans*. Fungal Genet Biol. 131:103241. doi:10.1016/j.fgb.2019.103241. 31220607

[jkaf071-B10] Fan Y, Lin X. 2018. Multiple applications of a transient CRISPR-Cas9 coupled with electroporation (TRACE) system in the *Cryptococcus neoformans* species complex. Genetics. 208(4):1357–1372. doi:10.1534/genetics.117.300656. 29444806 PMC5887135

[jkaf071-B11] Finkel JS, Yudanin N, Nett JE, Andes DR, Mitchell AP. 2011. Application of the systematic “DAmP” approach to create a partially defective *C. albicans* mutant. Fungal Genet Biol. 48(11):1056–1061. doi:10.1016/j.fgb.2011.07.005. 21820070 PMC3185220

[jkaf071-B12] Fisher MC, Denning DW. 2023. The WHO fungal priority pathogens list as a game-changer. Nat Rev Microbiol. 21(4):211–212. doi:10.1038/s41579-023-00861-x. 36747091 PMC9901396

[jkaf071-B13] Fu C, Robbins N, Cowen LE. 2024. Adaptation of the tetracycline-repressible system for modulating the expression of essential genes in *Cryptococcus neoformans*. bioRxiv 626100. 10.1101/2024.11.29.626100, preprint: not peer reviewed. PMC12108051 40310102

[jkaf071-B14] Giaever G, Chu AM, Ni L, Connelly C, Riles L, Véronneau S, Dow S, Lucau-Danila A, Anderson K, André B, et al 2002. Functional profiling of the *Saccharomyces cerevisiae* genome. Nature. 418(6896):387–391. doi:10.1038/nature00935. 12140549

[jkaf071-B15] Gietz RD, Schiestl RH. 2007. High-efficiency yeast transformation using the LiAc/SS carrier DNA/PEG method. Nat Protoc. 2(1):31–34. doi:10.1038/nprot.2007.13. 17401334

[jkaf071-B16] Goins CL, Gerik KJ, Lodge JK. 2006. Improvements to gene deletion in the fungal pathogen *Cryptococcus neoformans*: absence of Ku proteins increases homologous recombination, and co-transformation of independent DNA molecules allows rapid complementation of deletion phenotypes. Fungal Genet Biol. 43(8):531–544. doi:10.1016/j.fgb.2006.02.007. 16714127

[jkaf071-B17] Hu X, Yang P, Chai C, Liu J, Sun H, Wu Y, Zhang M, Zhang M, Liu X, Yu H. 2023. Structural and mechanistic insights into fungal β-1,3-glucan synthase *FKS1*. Nature. 616(7955):190–198. doi:10.1038/s41586-023-05856-5. 36949198 PMC10032269

[jkaf071-B18] Huang MY, Joshi MB, Boucher MJ, Lee S, Loza LC, Gaylord EA, Doering TL, Madhani HD. 2022. Short homology-directed repair using optimized Cas9 in the pathogen *Cryptococcus neoformans* enables rapid gene deletion and tagging. Genetics. 220(1):iyab180. doi:10.1093/genetics/iyab180. 34791226 PMC8733451

[jkaf071-B19] Ianiri G, Idnurm A. 2015. Essential gene discovery in the basidiomycete *Cryptococcus neoformans* for antifungal drug target prioritization. mBio. 6(2):e02334-14. doi:10.1128/mBio.02334-14. 25827419 PMC4453551

[jkaf071-B20] Kolodrubetz D, Rykowski MC, Grunstein M. 1982. Histone H2A subtypes associate interchangeably in vivo with histone H2B subtypes. Proc Natl Acad Sci U S A. 79(24):7814–7818. doi:10.1073/pnas.79.24.7814. 6760203 PMC347439

[jkaf071-B21] Li S, Prasanna X, Salo VT, Vattulainen I, Ikonen E. 2019. An efficient auxin-inducible degron system with low basal degradation in human cells. Nat Methods. 16(9):866–869. doi:10.1038/s41592-019-0512-x. 31451765

[jkaf071-B22] Lin J, Fan Y, Lin X. 2020. Transformation of *Cryptococcus neoformans* by electroporation using a transient CRISPR-cas9 expression (TRACE) system. Fungal Genet Biol. 138:103364. doi:10.1016/j.fgb.2020.103364. 32142753 PMC7153975

[jkaf071-B23] Milholland KL, Gregor JB, Hoda S, Píriz-Antúnez S, Dueñas-Santero E, Vu BG, Patel KP, Moye-Rowley WS, Vázquez de Aldana CR, Correa-Bordes J, et al 2023. Rapid, efficient auxin-inducible protein degradation in *Candida* pathogens. mSphere. 8(5):e0028323. doi:10.1128/msphere.00283-23. 37594261 PMC10597344

[jkaf071-B24] Missall TA, Lodge JK. 2005. Thioredoxin reductase is essential for viability in the fungal pathogen *Cryptococcus neoformans*. Eukaryot Cell. 4(2):487–489. doi:10.1128/EC.4.2.487-489.2005. 15701811 PMC549343

[jkaf071-B25] Mouyna I, Henry C, Doering TL, Latgé J-P. 2004. Gene silencing with RNA interference in the human pathogenic fungus *Aspergillus fumigatus*. FEMS Microbiol Lett. 237(2):317–324. doi:10.1016/j.femsle.2004.06.048. 15321679

[jkaf071-B26] Muhlrad D, Parker R. 1999. Aberrant mRNAs with extended 3′ UTRs are substrates for rapid degradation by mRNA surveillance. RNA. 5(10):1299–1307. doi:10.1017/s1355838299990829. 10573121 PMC1369852

[jkaf071-B27] Nabet B, Roberts JM, Buckley DL, Paulk J, Dastjerdi S, Yang A, Leggett AL, Erb MA, Lawlor MA, Souza A, et al 2018. The dTAG system for immediate and target-specific protein degradation. Nat Chem Biol. 14(5):431–441. doi:10.1038/s41589-018-0021-8. 29581585 PMC6295913

[jkaf071-B28] Nalley MJ, Banerjee S, Huang MY, Madhani HD. 2025. Near 100% efficient homology-dependent genome engineering in the human fungal pathogen *Cryptococcus neoformans*. bioRxiv 638277. 10.1101/2025.02.14.638277, preprint: not peer reviewed. PMC12341884 40460280

[jkaf071-B29] Nishimura K, Fukagawa T, Takisawa H, Kakimoto T, Kanemaki M. 2009. An auxin-based degron system for the rapid depletion of proteins in nonplant cells. Nat Methods. 6(12):917–922. doi:10.1038/nmeth.1401. 19915560

[jkaf071-B30] O’Meara TR, Veri AO, Ketela T, Jiang B, Roemer T, Cowen LE. 2015. Global analysis of fungal morphology exposes mechanisms of host cell escape. Nat Commun. 6(1):6741. doi:10.1038/ncomms7741. 25824284 PMC4382923

[jkaf071-B31] Ory JJ, Griffith CL, Doering TL. 2004. An efficiently regulated promoter system for *Cryptococcus neoformans* utilizing the CTR4 promoter. Yeast. 21(11):919–926. doi:10.1002/yea.1139. 15334556

[jkaf071-B32] Park Y-N, Morschhäuser J. 2005. Tetracycline-inducible gene expression and gene deletion in *Candida albicans*. Eukaryot Cell. 4(8):1328–1342. doi:10.1128/EC.4.8.1328-1342.2005. 16087738 PMC1214539

[jkaf071-B33] Peterson PP, Choi J-T, Fu C, Cowen LE, Sun S, Bahn Y-S, Heitman J. 2024. The *Cryptococcus neoformans* STRIPAK complex controls genome stability, sexual development, and virulence. PLoS Pathog. 20(11):e1012735. doi:10.1371/journal.ppat.1012735. 39561188 PMC11614259

[jkaf071-B34] Prozzillo Y, Fattorini G, Santopietro MV, Suglia L, Ruggiero A, Ferreri D, Messina G. 2020. Targeted protein degradation tools: overview and future perspectives. Biology (Basel). 9(12):421. doi:10.3390/biology9120421. 33256092 PMC7761331

[jkaf071-B35] Prusty R, Grisafi P, Fink GR. 2004. The plant hormone indoleacetic acid induces invasive growth in *Saccharomyces cerevisiae*. Proc Natl Acad Sci U S A. 101(12):4153–4157. doi:10.1073/pnas.0400659101. 15010530 PMC384710

[jkaf071-B36] Rajasingham R, Smith RM, Park BJ, Jarvis JN, Govender NP, Chiller TM, Denning DW, Loyse A, Boulware DR. 2017. Global burden of disease of HIV-associated cryptococcal meningitis: an updated analysis. Lancet Infect Dis. 17(8):873–881. doi:10.1016/S1473-3099(17)30243-8. 28483415 PMC5818156

[jkaf071-B37] Ram Y, Dellus-Gur E, Bibi M, Karkare K, Obolski U, Feldman MW, Cooper TF, Berman J, Hadany L. 2019. Predicting microbial growth in a mixed culture from growth curve data. Proc Natl Acad Sci U S A. 116(29):14698–14707. doi:10.1073/pnas.1902217116. 31253703 PMC6642348

[jkaf071-B38] Rappleye CA, Engle JT, Goldman WE. 2004. RNA interference in *Histoplasma capsulatum* demonstrates a role for alpha-(1,3)-glucan in virulence. Mol Microbiol. 53(1):153–165. doi:10.1111/j.1365-2958.2004.04131.x. 15225311

[jkaf071-B39] Roemer T, Jiang B, Davison J, Ketela T, Veillette K, Breton A, Tandia F, Linteau A, Sillaots S, Marta C, et al 2003. Large-scale essential gene identification in *Candida albicans* and applications to antifungal drug discovery. Mol Microbiol. 50(1):167–181. doi:10.1046/j.1365-2958.2003.03697.x. 14507372

[jkaf071-B40] Ross RL, Santiago-Tirado FH. 2024. Advanced genetic techniques in fungal pathogen research. mSphere. 9(4):e0064323. doi:10.1128/msphere.00643-23. 38470131 PMC11036804

[jkaf071-B41] Schuldiner M, Collins SR, Thompson NJ, Denic V, Bhamidipati A, Punna T, Ihmels J, Andrews B, Boone C, Greenblatt JF, et al 2005. Exploration of the function and organization of the yeast early secretory pathway through an epistatic miniarray profile. Cell. 123(3):507–519. doi:10.1016/j.cell.2005.08.031. 16269340

[jkaf071-B42] Segal ES, Gritsenko V, Levitan A, Yadav B, Dror N, Steenwyk JL, Silberberg Y, Mielich K, Rokas A, Gow NAR, et al 2018. Gene essentiality analyzed by in vivo transposon mutagenesis and machine learning in a stable haploid isolate of *Candida albicans*. mBio. 9(5):e02048-18. doi:10.1128/mBio.02048-18. 30377286 PMC6212825

[jkaf071-B43] Sepers JJ, Verstappen NHM, Vo AA, Ragle JM, Ruijtenberg S, Ward JD, Boxem M. 2022. The mIAA7 degron improves auxin-mediated degradation in *Caenorhabditis elegans*. G3 (Bethesda). 12(10):jkac222. doi:10.1093/g3journal/jkac222. 36029236 PMC9526053

[jkaf071-B44] Sikorski RS, Hieter P. 1989. A system of shuttle vectors and yeast host strains designed for efficient manipulation of DNA in *Saccharomyces cerevisiae*. Genetics. 122(1):19–27. doi:10.1093/genetics/122.1.19. 2659436 PMC1203683

[jkaf071-B45] Sladky VC, Strong MA, Tapias-Gomez D, Jewett CE, Drown CG, Scott PM, Holland AJ. 2024. The AID2 system offers a potent tool for rapid, reversible, or sustained degradation of essential proteins in live mice. bioRxiv 597287. 10.1101/2024.06.04.597287, preprint: not peer reviewed.

[jkaf071-B46] Suski JM, Ratnayeke N, Braun M, Zhang T, Strmiska V, Michowski W, Can G, Simoneau A, Snioch K, Cup M, et al 2022. CDC7-independent G1/S transition revealed by targeted protein degradation. Nature. 605(7909):357–365. doi:10.1038/s41586-022-04698-x. 35508654 PMC9106935

[jkaf071-B47] Tanaka S, Miyazawa-Onami M, Iida T, Araki H. 2015. iAID: an improved auxin-inducible degron system for the construction of a “tight” conditional mutant in the budding yeast *Saccharomyces cerevisiae*. Yeast. 32(8):567–581. doi:10.1002/yea.3080. 26081484

[jkaf071-B48] Thompson JR, Douglas CM, Li W, Jue CK, Pramanik B, Yuan X, Rude TH, Toffaletti DL, Perfect JR, Kurtz M. 1999. A glucan synthase *FKS1* homolog in *Cryptococcus neoformans* is single copy and encodes an essential function. J Bacteriol. 181(2):444–453. doi:10.1128/JB.181.2.444-453.1999. 9882657 PMC93397

[jkaf071-B49] van Opijnen T, Bodi KL, Camilli A. 2009. Tn-seq: high-throughput parallel sequencing for fitness and genetic interaction studies in microorganisms. Nat Methods. 6(10):767–772. doi:10.1038/nmeth.1377. 19767758 PMC2957483

[jkaf071-B50] Wensing L, Shapiro RS. 2022. Design and generation of a CRISPR interference system for genetic repression and essential gene analysis in the fungal pathogen *Candida albicans*. Methods Mol Biol. 2377:69–88. doi:10.1007/978-1-0716-1720-5_4. 34709611

[jkaf071-B51] Yan Z, Costanzo M, Heisler LE, Paw J, Kaper F, Andrews BJ, Boone C, Giaever G, Nislow C. 2008. Yeast Barcoders: a chemogenomic application of a universal donor-strain collection carrying bar-code identifiers. Nat Methods. 5(8):719–725. doi:10.1038/nmeth.1231. 18622398

[jkaf071-B52] Yesbolatova A, Saito Y, Kitamoto N, Makino-Itou H, Ajima R, Nakano R, Nakaoka H, Fukui K, Gamo K, Tominari Y, et al 2020. The auxin-inducible degron 2 technology provides sharp degradation control in yeast, mammalian cells, and mice. Nat Commun. 11(1):5701. doi:10.1038/s41467-020-19532-z. 33177522 PMC7659001

