# Supplementary material for: An auxin-inducible degron system for conditional mutation in the fungal meningitis pathogen Cryptococcus neoformans
Source: G3 (Bethesda). 2025 Apr 7;15(6):jkaf071. doi: 10.1093/g3journal/jkaf071 (PMC12134991; doi:10.1093/g3journal/jkaf071)
Supplement: jkaf071_Supplementary_Data [file jkaf071_supplementary_data.zip › Figure_S1_G3-2025-405820.pdf]

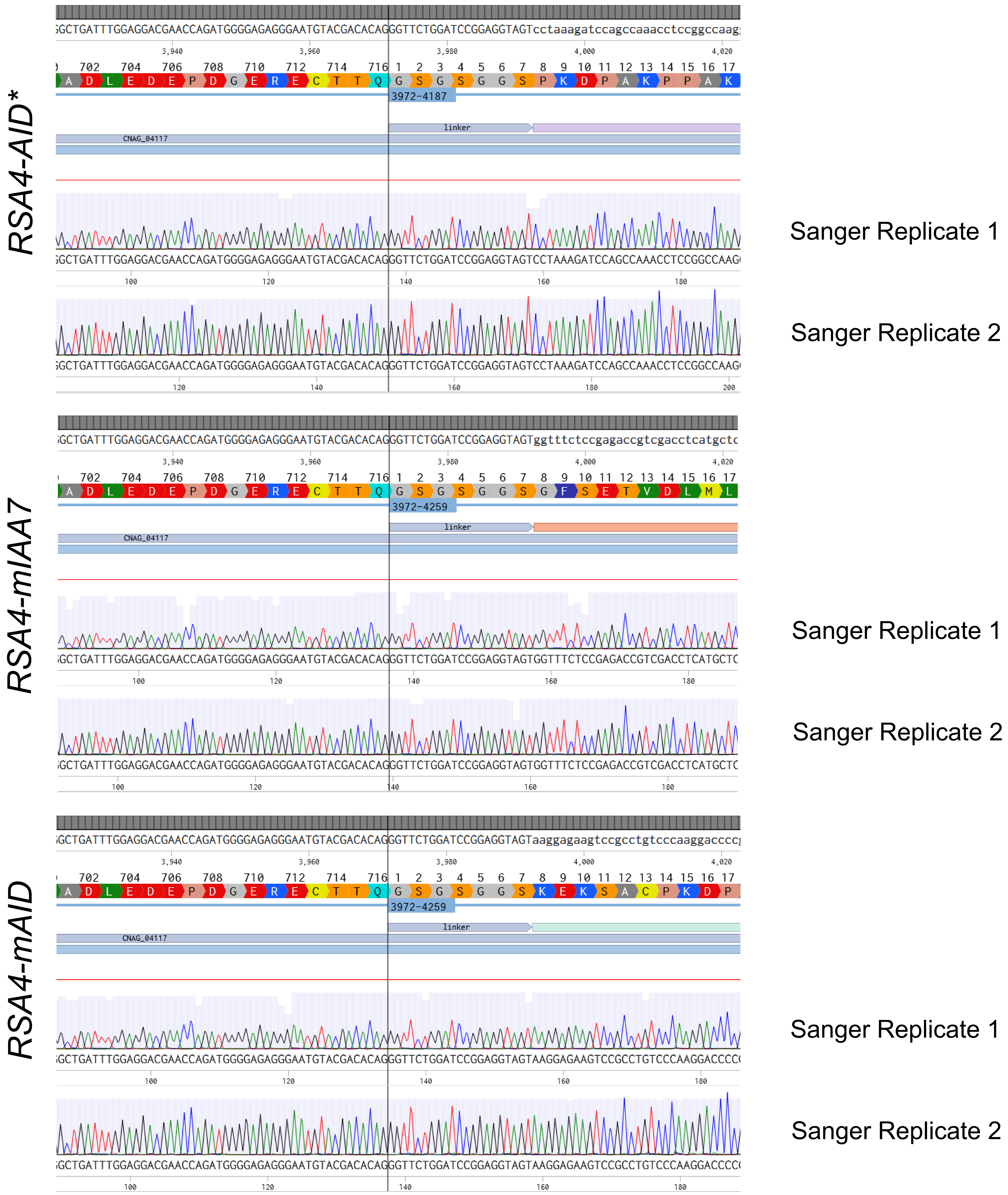

Figure S1. Alignment of Sanger sequencing of *RSA4* tag junctions. Images are screenshots from alignments analyzed in Benchling. Two replicates were sequenced for each strain, one for each primer used to amplify the junction locus to sequence in both directions.
