# Supplementary material for: An auxin-inducible degron system for conditional mutation in the fungal meningitis pathogen Cryptococcus neoformans
Source: G3 (Bethesda). 2025 Apr 7;15(6):jkaf071. doi: 10.1093/g3journal/jkaf071 (PMC12134991; doi:10.1093/g3journal/jkaf071)
Supplement: jkaf071_Supplementary_Data [file jkaf071_supplementary_data.zip › Figure_S2_G3-2025-405820.pdf]

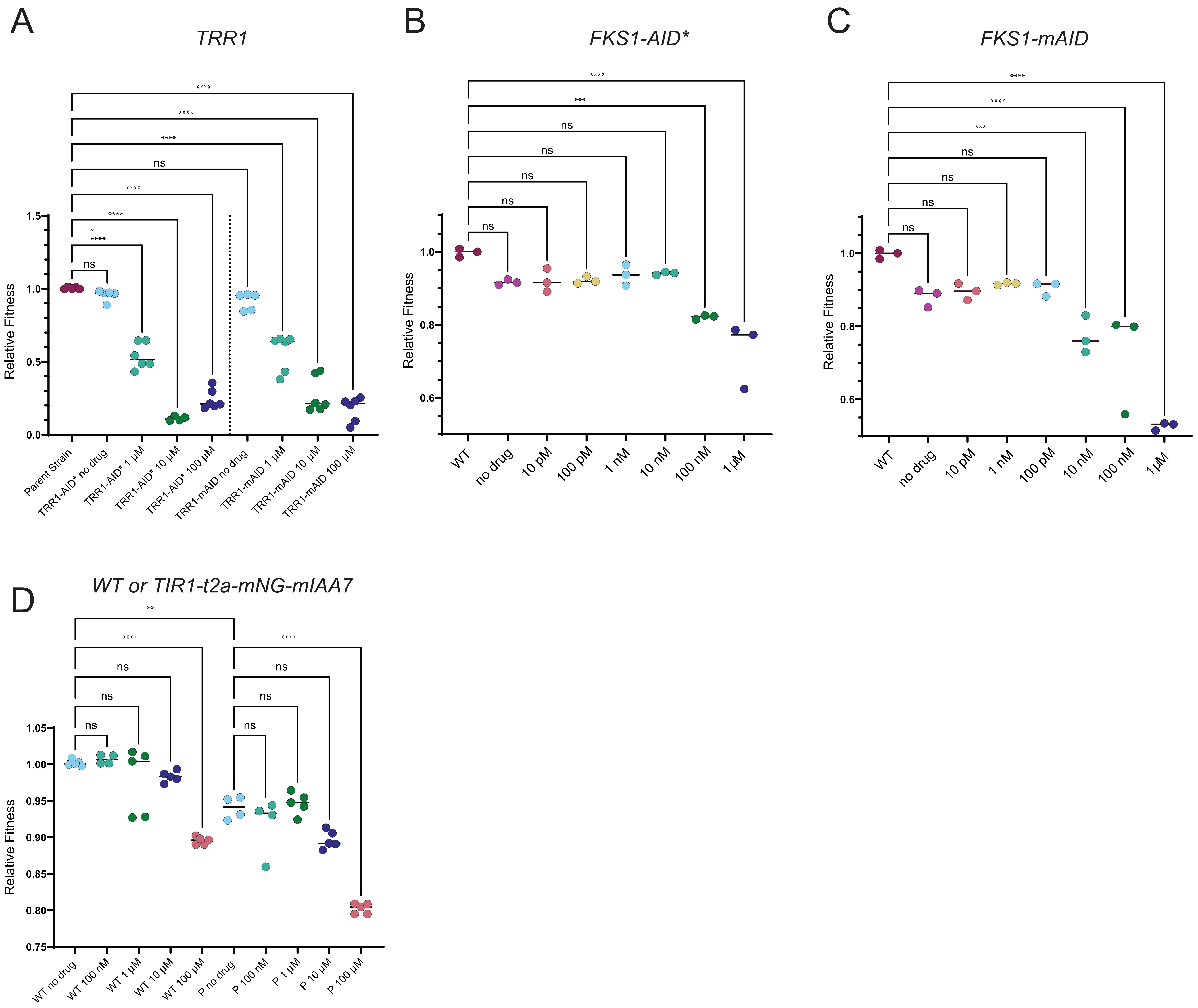

Figure S2

Relative fitness to a wild type liquid growth culture was estimated using Curveball software (Ram et al, 2019) and plotted for each growth curve replicate and condition. Asterisks denote statistically significant comparisons (* P < 0.05, ** P < 0.01, *** P < 0.001, **** P < 0.0001) as determined by Dunnett's multiple comparisons test for panels A, B, and C, or Tukey's multiple comparison test for panel D. Not all comparisons are shown in panel D. **A.** Corresponds to Figure 4A, 4B. Relative fitness for TRR1-AID* and TRR1-mAID strains across a range of 5-Ph-IAA concentrations. **B.** Corresponds to Figure 4C. Relative fitness for FKS1-AID* across a range of 5-Ph-IAA concentrations. **C.** Corresponds to Figure 4D. Relative fitness for FKS1-mAID across a range of 5-Ph-IAA concentrations. **D.** Corresponds to Figure 4E, 4F. Relative fitness for either wild type or TIR1-t2a-mNG-mIAA7 expressing strains (denoted as P on x axis) across a range of 5-Ph-IAA concentrations. Curves were not fitted to 1 mM samples as no growth was observed.
